# The clinical value of glycosylated hemoglobin level in newly diagnosed ketosis-prone type 2 diabetes

**DOI:** 10.3389/fendo.2023.1244008

**Published:** 2023-11-10

**Authors:** Rui Min, Yancheng Xu, Bocheng Peng

**Affiliations:** ^1^ Department of Geriatrics, Wuhan Fourth Hospital, Wuhan, Hubei, China; ^2^ Department of Endocrinology, Zhongnan Hospital, Wuhan University, Wuhan, Hubei, China; ^3^ Department of Pain, Wuhan Fourth Hospital, Wuhan, Hubei, China

**Keywords:** glycosylated hemoglobin, ketosis-prone type 2 diabetes, type 2 diabetes mellitus, ketosis, insulin function

## Abstract

**Objective:**

To evaluate the clinical value of glycosylated hemoglobin (HbA1c) in newly diagnosed ketosis-prone type 2 diabetes (KPD).

**Methods:**

A total of 330 patients with newly diagnosed type 2 diabetes (T2DM) hospitalized in our department with an average age of 48.72 ± 13.07 years old were selected and divided into T2DM group (193 cases) and KPD group (137 cases) according to whether they were combined with ketosis. According to the quartile level of HbA1c, they were divided into group A (HbA1c < 8.90%, 84 cases), group B (8.90%≤HbA1c < 10.70%, 86 cases), group C (10.70%≤HbA1c ≤ 12.40%, 85 cases) and group D (HbA1c > 12.40%, 75 cases). The general clinical features, laboratory indicators and islet function of each group were compared. Spearman correlation analysis was used to explore the correlation between HbA1c and β- Hydroxybutyric acid (β- HB) and islet function. ROC curve was used to analyze the sensitivity and specificity of HbA1c in diagnosing KPD, and the optimal tangent point was obtained.

**Results:**

HbA1c, β-HB, FFA, RBG, insulin dosage, GSP, OGTT (0, 0.5, 1, 2, 3h) in KPD group were significantly higher than those in T2DM group (P< 0.001). HDL-C, IRT (0, 0.5, 1, 2, 3h), HOMA-β, HOMA-IR, HOMA-IS, ΔC30/ΔG30, AUC _insulin_ were significantly lower than those in T2DM group (P< 0.001). With the increase of HbA1c level, the incidence of ketosis, β-HB, FFA and insulin dosage increased, while IRT (0, 0.5, 1, 2, 3h), ΔC30/ΔG30, AUC _insulin_, HOMA-β and HOMA-IS decreased accordingly (P< 0.001). In all newly diagnosed T2DM patients, Spearman correlation analysis showed that HbA1c was positively correlated with β-HB (r=0.539, P < 0.001), and was negatively correlated with HOMA-β (r=-0.564, P < 0.001), HOMA-IS (r=-0.517, P < 0.01, P < 0.001), HOMA-IR (r=-0.177, P < 0.001), ΔC30/ΔG30 (r=-0.427, P < 0.01) and AUC _insulin_ (r=-0.581, P < 0.001). In ROC curve analysis, the optimal threshold for the diagnosis of KPD was 10.15%, Youden index was 0.616, area under the curve (AUC) was 0.882, sensitivity = 92.70%, specificity = 70.50%.

**Conclusion:**

In newly diagnosed T2DM patients, if HbA1c > 10.15%, it is more likely to develop KPD. Monitoring HbA1c level is conducive to timely detection of high-risk individuals with KPD and taking appropriate measures to prevent the occurrence and development of the disease.

## Introduction

Among the acute complications of diabetes, ketosis or ketoacidosis (DKA) is a serious medical emergency which may lead to serious complications or even fatal consequences if not managed in time. It is usually considered to be the typical feature and first manifestation of Type 1 diabetes mellitus (T1DM) which is caused by the destruction of islet beta cells due to autoimmunity (1). However, in recent decades, research has found that more and more patients who have not found the classic autoimmune phenotype of T1DM are also often characterized by ketosis at the time of initial diagnosis, which has led to the recognition of a new atypical form of diabetes called Ketosis-prone type 2 diabetes (KPD) (2).

First reported in the U.S. literature in 1987, KPD is characterized by acute and severe hyperglycemia (blood glucose usually higher than 500 mg/dL and mean HbA1c> 10%) with ketosis similar to T1DM, but distinct from classic T1DM, it lacks autoimmune markers for islet beta cells (3). Over the past few decades, KPD has attracted increasing attention around the world and its incidence has been found to be on the rise (4). KPD is thought to account for 25% to 50% of African Americans and Hispanics with newly diagnosed diabetic ketoacidosis (5). Although most cases have been reported in Africans and African Americans in the United States, So far, such cases have been reported in different ethnic groups around the world including Japanese, Chinese, Hispanic, and white populations (3, 6–9). Regarding the prevalence of KPD, the perception of low prevalence of KPD among Asians may need to be changed, as a Korean study showed that 42% of all newly diagnosed T2DM patients had DKA (7)and in a Chinese study, KPD was diagnosed in 38% of patients with new-onset type 2 diabetes (8).

KPD patients are usually middle-aged men who are overweight or obese and have a strong family history of T2DM (10). The increased incidence of KPD may be related to the increased speed of life, lack of physical activity, lack of regular healthy diet and reduced exercise (11). In addition, the progress of examination methods also has a certain correlation with the increased incidence of KPD. The onset of ketosis may be related to the transient acute insulin deficiency on the basis of the underlying severe insulin resistance state. In the acute stage of KPD onset, the suddenly rising levels of glucose and free fatty acids (FFA) will lead to severe and persistent glycolipid toxicity (12). Continuous overstimulation of pancreatic beta cells is triggered, which further induces oxidative stress and mitochondrial dysfunction of pancreatic beta cells, resulting in dysfunction, failure or even death of beta cells. If the acute dysfunction of glucose and lipid metabolism in patients is not controlled in a timely and effective manner, it will lead to higher blood glucose due to the reduced function and activity of beta cells, thus forming a vicious cycle (13).

It is worth noting that KPD patients, after several months of initial short-term intensive insulin therapy to correct hyperglycemia, beta cell function will gradually recover from the initial decompensated state, with significant improvements in insulin sensitivity (14). Patients can safely discontinue exogenous insulin therapy and maintain nearly normal good blood glucose control by relying solely on diet, lifestyle changes and/or oral hypoglycemic drugs, with a small number of patients suffering from recurrent ketosis (15).

Different from the typical T1DM, KPD patients usually have no clear inducement and obvious clinical symptoms at the onset of the disease, thus the prevalence of KPD has been greatly underestimated. However, KPD patients have a rapid onset, and they have a fairly high blood glucose level at the first visit, and the function of islet beta cells is seriously damaged, which is manifested by the significant reduction of C-peptide level (11). If ketosis is not judged and treated in time and thus accelerates the vicious cycle of glucolipid toxicity, it may lead to more serious consequences, such as acidosis, coma and even death (16).

Given its serious harm and potential adverse events, rapid diagnosis and early and timely hypoglycemic treatment are necessary for ketosis in clinical practice. At the same time, clinical work also urgently needs a method to recognize and prevent the occurrence of DK at an early stage, so as to reduce the incidence of diabetes-related emergencies and adverse events and improve the long-term quality of life of patients.

HbA1c plays an indispensable role in the management and control of diabetes. It is the gold standard to judge the long-term blood glucose control status, reflecting the average range of blood glucose levels over the past 2 to 3 months (17). Relevant retrospective studies found that almost all DKA patients had significantly increased HbA1c upon admission. Although DKA is considered to be an acute metabolic complication of diabetes, study shows that DKA is likely to occur in the context of long-term chronic hyperglycemia, suggesting that improving long-term glucose control in known diabetes patients may reduce the occurrence of DKA (18).

According to previous domestic and foreign studies, HbA1c, as a special predictor, has been widely used in the chronic complications of microvascular and macrovascular diseases of diabetes (19, 20). At present, the scope of use of HbA1c has been extended to the diagnosis of diabetes and the detection of diabetic retinopathy, and relevant reports have determined the diagnostic critical value (21).

At present, studies have shown that DKA in type 1 diabetes patients is associated with long-term elevated HbA1c levels, while the relationship between KPD and HbA1c levels is insufficient (22). This study hypothesized that there was a correlation between KPD and HbA1c, discussed the influence of HbA1c level on the occurrence of KPD in newly diagnosed T2DM patients, and quantitatively analyzed the relationship between HbA1c level and β-HB and islet function, aiming to preliminatively monitor the occurrence of KPD early through HbA1c and provide clinical application value for prevention and treatment of KPD.

## Materials and methods

### Study design and participants

We conducted this retrospective case analysis study based on data collected from our previous studies and data collected from open source databases (23). The G*Power 3.1.9.2 software was used to calculate the sample size for prior power analysis, and the required sample size was 210 participants when the effect size f=0.5, α=0.05, 1-β=0.95. This cross-sectional retrospective clinical study included 330 newly diagnosed T2DM patients hospitalized from January 2019 to December 2022 who tested negative for islet autoantibodies and had not received any medication, including 249 males (75.45%) and 81 females (24.55%), aged 21-77 years, with an average age of 48.72 ± 13.07 years. According to the presence or absence of ketosis, the patients were divided into type 2 diabetes without ketosis group (T2DM group, 193 cases, 58.48%) and T2DM with ketosis group (KPD group, 137 cases, 41.52%). At last, G*Power software was used to conduct a posterior power analysis of the statistical efficacy of the sample size included in this study. When the effect size d=0.5, α=0.05, 1-β=0.994 was obtained, it was proved that this sample size could achieve good statistical efficacy. According to the HbA1c quartile level, the included subjects were divided into group A (HbA1c < 8.90%, 84 cases), group B (8.90%≤HbA1c<10.70%, 86 cases), group C (10.70%≤HbA1c ≤ 12.40%, 85 cases), and group D (HbA1c > 12.40%, 75 cases). According to the definition and criteria for diabetes set by the America Diabetes Association (ADA) of 2019, T2DM was defined by fasting blood glucose (FBG)≥7.0 mmol/L and/or 2h-postprandial plasma glucose (2h PG) ≥11.1mmol/L during a 75 g oral glucose tolerance test. The inclusion criteria were as follows (1): Newly diagnosed with untreated type 2 diabetes, the course of disease is within half a year; (2) KPD patients with ketosis were defined as positive blood ketones (β-hydroxybutyric acid > 0.30 mmol/L) and/or moderate to heavy urine ketones (urine ketones ≥ (2+)), without glutamic acid decarboxylase antibody (GAD) and insulin autoantibody (IAA), and there were no obvious inducement, no clinical manifestations of ketoacidosis, nor did they meet the diagnostic criteria for ketoacidosis; T2DM without ketosis were defined as diabetes with neither diabetic ketosis (β-hydroxybutyric acid ≤ 0.30mmol/L and ketone body (-)) nor GAD or IAA autoantibodies. The exclusion criteria were as follows: (1) Patients with severe infection, acute myocardial infarction, acute cerebral infarction, kidney injury, delirium, seizures, acute alcohol intoxication, pregnancy diabetes, severe trauma, malignant tumor, surgery, corticoid therapy, etc., which might result in ketosis; (2) Patients with diabetic ketoacidosis; (3) Patients who have taken any hypoglycemic drugs or other drugs that may affect glucose and lipid metabolism; (4) Patients with severe liver function or renal insufficiency; (5) Secondary diabetes, such as pancreatic exocrine diseases and other endocrine diseases; (6) Patients with incomplete clinical data. The study complies with the Declaration of Helsinki and has been approved by the Ethics Committee of Wuhan Fourth Hospital (KY2023-067-01).

### Data collection

(1) General clinical data of patients were collected through a comprehensive and careful consultation by professional doctors, including basic information such as age, sex, present and past medical history. Nurses with standardized training underwent a simple physical examination, including the measurement of height (m) and body weight (kg) of all patients, from which the body mass index (BMI) = kg/m²was measured. When the patient first visited a doctor, the random blood glucose (RBG) of the finger or vein, blood β-hydroxybutyric acid (β- HB), urine routine, arterial blood gas analysis and other indexes were urgently checked.

(2) The inpatients were instructed to keep fasting for at least eight hours before exsanguinate assay in the next morning. The venous blood samples collected including GAD-Ab, IAA-Ab, glycosylated serum protein (GSP), HbA1c, total triglycerides (TG), total cholesterol (TC), high-density lipoprotein cholesterol (HDL-C), low-density lipoprotein cholesterol (LDL-C), lipoprotein a [LP (a)] and FFA, etc., among which GDA-AB and IAA-Ab were determined by qualitative ELISA, FPG was determined by serum oxidase, GSP was determined by enzymatic method, HbA1c was determined by high performance liquid chromatography, and TC, TG, HDL-C, LDL-C, and FFA were determined by standard enzymes. All indexes were measured on Beckman AU5400 automatic analyzer according to standard laboratory methods.

(3) In T2DM patients without ketosis, 75g glucose tolerance test (standard OGTT test) and insulin release test (IRT) were performed in the morning of the second day after hospitalization, while patients with KPD first took insulin hypoglycemic and related measures, and then performed OGTT test and IRT test within 24 hours after ketosis was corrected. The subject patients fasted overnight from the previous day. From 7 am to 9 am, blood was drawn for fasting plasma glucose (OGTT0h) and fasting insulin (IRT0h) under the condition of fasting and then 75 g anhydrous glucose dissolved in 200-300ml water was taken within 5 minutes. KPD patients were replaced with steamed bread containing 100g flour and finished eating within 10-15 minutes. Blood samples were collected from the anterior cubitus vein at intervals of 30, 60, 120, and 180 minutes to measure blood glucose concentration (OGTT 0.5h, OGTT 1h, OGTT 2h, OGTT 3h) and serum insulin concentration (IRT 0.5h, IRT 1h, IRT 2h, IRT 3h). The 5-phase blood glucose was measured by glucose oxidase method, and the level of serum 5-phase insulin level was determined by radioligand method established by radioimmunoassay. The blood samples should be submitted for examination by professionals as soon as possible after blood collection.

(4) During the patient’s hospitalization, the specific daily insulin adjusted dose was recorded in detail. Go through the discharge according to whether the blood glucose level was stable, and the daily insulin dose required for subcutaneous injection at discharge was recorded at the same time.

### Assessment of islet function

Insulin secretion index (HOMA-β) = 20×FINS (uIU/L)/[FPG (mmol/L) -3.5] and insulin resistance index (HOMA-IR=FBG (mmol/L)×FINS (uIU/L)/22.5 were evaluated according to homeostasis model. The ratio ΔI30/ΔG30 of insulin increment to glucose increment at 30min after glucose loading in OGTT test was used to evaluate the early phase (phase 1) secretion of pancreatic β-cells. The area under the OGTT insulin curve AUC_insulin_ = (I0h+2×I0.5h+3×I1h+4×I2h+2×I3h)/4 was used to evaluate the late phase (phase 2) secretion of islet beta cells, and the trapezoidal method was used to calculate the AUC value.

### Statistical analysis

This study utilized IBM SPSS 22.0 software and MedCalc software for data processing and statistical analysis. G*Power 3.1.9.2 software was used to perform a prior and a posterior statistical analysis. The Kolmogorov–Smirnov test was used to determine the index’s distribution. The continuous measurement data subject to normal distribution and non-normal distribution are represented by “mean ± standard deviation (x ± s)” and “median (lower Quartile, upper Quartile) [(M (P25, P75)]” respectively. The comparison of data between two groups was conducted using two independent sample t-tests and wilcoxon rank sum tests, while the comparison of data between multiple groups was conducted using Single factor analysis of variance and Kruskal Wallis H-test (Bonferroni method was used to correct for pairwise comparisons between groups). Counting data adopted χ 2 test, expressed as a rate (%), and two-way ordered data were analyzed by trend chi-square test. Correlation analysis between variables uses Spearman correlation analysis to determine the hierarchical correlation between variables. Receiver operating characteristic (ROC) curve was used to analyze the sensitivity and specificity of different HbA1c levels for the occurrence of KPD, and the area under the ROC curve was calculated. MedCalc software was used to plot ROC curves and perform comparisons between multiple ROC curves and calculate P-values. The optimal HbA1c threshold for the diagnosis of KPD was determined by Youden index (sensitivity + specificity -1), so as to identify high-risk groups of KPD early. A two-tailed p-value less than 0.05 was considered as statistically significant and the adjusted test level for data comparison among the four groups was P<0.0125.

## Results

### Baseline clinical characteristics of participants

Among the total 330 patients included in this study, 193 were in the T2DM group, accounting for 58.48% of the total, and 137 were in the KPD group, accounting for 41.52% of the total. Compared with T2DM group, KPD group had a younger onset age, a higher proportion of male patients, and a higher dosage of insulin when blood glucose was stabilized. RBG, HbA1c (12.67% vs 9.32%), β-HB, FFA, GSP, OGTT (0, 0.5, 1, 2, 3h) were significantly higher than those in T2DM group (P< 0.05). HDL-C, IRT (0, 0.5, 1, 2, 3h), HOMA-IR, HOMA-β, ΔC30/ΔG30 and AUC _insulin_ were significantly lower than those in T2DM group (P< 0.05), and there were no statistically significant differences between the two groups in BMI, TG, TC, LDL-C and LP (a) (P> 0.05) (**Table 1**).

**Table 1:** Comparison of clinical characteristics between the KPD group and the T2DM group [n (%), x ± s, M (P25,P75)].

	T2DM group (n=193)	KPD group (n=137)	T/Z/X^2^	P value
Age (years)	52.00 (42.00, 62.00)	46.00 (33.50, 55.00)	-4.301	<0.001
Gender (male/female)	135/58(69.95%)	114/23(83.21%)	7.611	0.006
BMI(kg/m2)	25.39 (23.78, 27.62)	24.93 (22.39, 28.08)	-1.313	0.189
Insulin dosage(u/d*kg)	0.15 (0.00, 0.45)	0.46 (0.34, 0.59)	-6.471	<0.001
RBG(mmol/L)	14.32 ± 3.70	21.86 ± 6.23	-13.764	<0.001
HbA1c(%)	9.32 ± 2.01	12.67 ± 2.22	-14.253	<0.001
β-HB(mmol/L)	0.12 (0.08, 0.17)	0.5(0.29, 1.21)	-12.740	<0.001
TC(mmol/L)	5.00 (4.35,5.68)	5.04 (4.35, 5.97)	-0.666	0.505
TG(mmol/L)	1.93 (1.37, 2.78)	1.94 (1.23, 2.87)	-0.288	0.773
HDL-C(mmol/L)	1.01 (0.85, 1.14)	0.92 (0.76, 1.15)	-2.700	0.007
LDL-C(mmol/L)	3.06 ± 0.90	3.05 ± 1.13	0.083	0.934
LP(a)(mg/L)	81.90(40.05,149.50)	77.80 (39.00, 182.50)	-0.373	0.709
FFA(ummol/L)	509.23(411.79,668.07)	613.87 (465.16, 791.49)	-3.897	<0.001
GSP(umol/L)	379.85 (320.80,468.81)	521.60 (451.05, 610.30)	-9.364	<0.001
OGTT0h(mmol/L)	8.92 (7.59, 11.25)	11.99 (9.00, 13.75)	-5.876	<0.001
OGTT0.5h(mmol/L)	14.18 ± 3.43	16.10 ± 3.84	-4.753	<0.001
OGTT1h(mmol/L)	17.99 ± 4.13	20.46 ± 4.22	-5.302	<0.001
OGTT2h(mmol/L)	18.53 ± 4.68	22.79 ± 4.37	-8.360	<0.001
OGTT3h(mmol/L)	15.06 ± 5.31	20.10 ± 4.56	-8.998	<0.001
IRT0h(mmol/L)	10.70 (6.68, 16.05)	5.74 (3.58, 10.11)	-7.420	<0.001
IRT0.5h(mmol/L)	18.90 (12.10, 28.40)	8.89 (5.40, 15.60)	-8.245	<0.001
IRT1h(mmol/L)	28.50 (16.60, 44.34)	12.10 (6.97, 20.30)	-9.096	<0.001
IRT2h(mmol/L)	34.80 (21.40, 56.15)	14.30 (9.14, 24.45)	-8.758	<0.001
IRT3h(mmol/L)	29.20 (16.90, 44.30)	12.80 (8.23, 22.00)	-8.409	<0.001
ΔC30/ΔG30	1.52(0.66, 3.24)	0.55 (0.22, 1.27)	-6.076	<0.001
AUC_insulin_(mmol/L)	85.18 (51.63, 132.20)	36.50 (23.94, 61.55)	-8.980	<0.001
HOMA - IR	4.32 (2.73, 6.64)	2.77(1.72, 4.70)	-5.253	<0.001
HOMA - β	41.46 (20.54, 68.40)	15.01(8.38, 27.45)	-8.440	<0.001

BMI, body mass index; RBG, random blood glucose; HbA1c, glycosylated hemoglobin A1c; β-HB, β-hydroxybutyric acid; TG, triglyceride; TC, total cholesterol; HDL-C, high density lipoprotein cholesterol; LDL-C, low density lipoprotein cholesterol; LP (a), lipoprotein a; FFA, free fatty acids; GSP, glycosylated serum protein; OGTT (0,0.5,1,2,3h), oral glucose tolerance test (0,0.5,1,2,3h); IRT (0,0.5,1,2,3h), Insulin release test(0,0.5,1,2,3h); ΔC30/ΔG30, insulin early phase secretion index; AUC insulin, area under the insulin curve; HOMA-IR, insulin resistance index; HOMA-β, insulin secretion index; HOMA-IS, Insulin sensitivity index.

### Comparison of ketosis incidence, β-HB, FFA and islet function at different HbA1c levels

**Figure 1:**
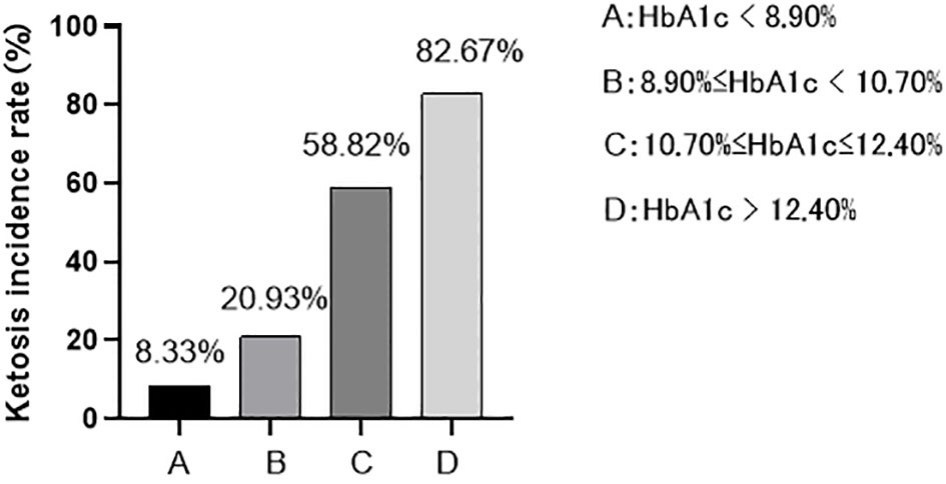
The incidence of ketosis at different HbA1c levels: As HbA1c levels increase, the incidence of ketosis also increases (8.33% < 20.93% < 58.82% < 82.67%).

According to the Quartile level of HbA1c, they were divided into group A (84 cases with HbA1c<8.90%), group B (86 cases with 8.90% ≤ HbA1c<10.70%), group C (85 cases with 10.70% ≤ HbA1c ≤ 12.40%), and group D (75 cases with HbA1c>12.40%). In this study, it was found that with the increase of HbA1c level, the incidence of ketosis also increased, and the difference was statistically significant (x2 trend =111.723, P < 0.001) (**Figure 1**). There were significant differences in β-HB, insulin dosage, FFA, IRT (0, 0.5, 1, 2, 3h), HOMA-β, HOMA-IR, ΔC30/ΔG30 and AUC _insulin_ among the four groups (P < 0.05). With the increase of HbA1c level, β-HB, insulin dosage and FFA increased successively, while IRT (0, 0.5, 1, 2, 3h), ΔC30/ΔG30, AUC _insulin_ and HOMA-β decreased successively and the concentration of β-HB in group D was significantly higher than that in groups A, B and C, and the concentration in group C was significantly higher than that in groups A and B (P<0.05). The insulin dosage in group D was significantly higher than that in groups A, B, and C, while group B and C were significantly higher than group A (P<0.05). The FFA concentration in group D was significantly higher than that in group A, and there was no statistically significant difference between the other groups (P>0.05). The IRT (0, 0.5, 1, and 3 h) and AUC _insulin_ levels in groups C and D were significantly lower than those in groups A and B (P<0.05). IRT2h, Δ C30/Δ G30, HOMA- β in group C and D was significantly lower than groups A and B, and Group B was significantly lower than Group A (P<0.05). With the increase of HbA1c level, HOMA-IR showed a trend of first increasing and then decreasing, and there was no significant difference between the other groups (P > 0.05) except for the difference between group B and group D (P < 0.05) (**Table 2**).

**Table 2:** Comparison of ketosis incidence, β-HB, FFA and islet function at different HbA1c levels [n (%), M (P25,P75)].

	Group A (n=84)	Group B (n=86)	Group C (n=85)	Group D (n=75)	X^2^_trend_/F value	P value
HbA1c(%)	<8.90	8.90-10.70	10.70-12.40	>12.40		
Ketosis(with/without)	7/77 (8.33%)	18/68 (20.93%)	50/35 (58.82%)	62/13(82.67%)	111.723	<0.001
β-HB(mmol/L)	0.11(0.08,0.17)^ab^	0.14(0.11,0.23)^ab^	0.25(0.12,0.48)^a^	0.75(0.22,2.29)	79.583	<0.001
Insulin dosage(u/d*kg)	0.00 (0.00, 0.30)^abc^	0.32 (0.00, 0.46)^a^	0.42 (0.22, 0.54)^a^	0.56 (0.40, 0.70)	104.39	<0.001
FFA(ummol/L)	510.16 (355.48, 663.99)^a^	546.71 (444.17, 688.34)	570.83 (439.03, 703.68)	587.10(462.57,754.90)	9.859	0.020
IRT0h(ummol/L)	11.62(6.63,17.25)^ab^	10.33(6.65,15.23)^ab^	7.72(4.50,10.45)	5.55(3.25,10.40)	40.998	<0.001
IRT0.5h(ummol/L)	24.60(13.37,42.85)^ab^	17.00(12.20,22.20)^ab^	9.69(7.03,16.45)	7.70 (4.96, 13.20)	83.166	<0.001
IRT1h(ummol/L)	41.15(22.45,71.63)^ab^	25.15(18.45,35.70)^ab^	13.60(8.68,20.50)	9.91(6.40,17.38)	109.844	<0.001
IRT2h(ummol/L)	46.00(28.50,97.40)^abc^	32.50(20.90,43.45)^ab^	17.40(11.05,29.80)	11.90(8.07,23.10)	101.724	<0.001
IRT3h(ummol/L)	36.25(19.92,57.82)^ab^	28.00(17.54,40.51)^ab^	15.02(10.90,22.40)	11.70(6.70,21.60)	81.256	<0.001
HOMA - β	55.31(30.82,92.46)^abc^	36.07(20.85,54.60)^ab^	18.27(10.16,28.43)	12.94(7.21,24.75)	91.548	<0.001
HOMA - IR	4.07(2.30,5.86)	4.70(2.73,6.60)^a^	3.67(2.28,6.00)	2.71(1.58,4.58)	13.616	0.003
ΔC30/ΔG30	2.81(1.02,5.10)^abc^	1.32(0.53,2.94)^ab^	0.65(0.32,1.23)	0.59(0.21,1.13)	53.696	<0.001
AUC_Insulin_(ummol/L)	116.65(66.41,194.38)^ab^	74.00(53.15,105.49)^ab^	43.70(28.31,68.44)	31.39(20.82,56.56)	99.296	<0.001

Compared with group D, ^a^P (adjusted) was < 0.05; Compared with group C, ^b^P (adjusted) was <0.05; Compared with group B, ^c^P (adjusted) was <0.05.

HbA1c, glycosylated hemoglobin A1c; β-HB, β-hydroxybutyric acid; FFA, free fatty acids; IRT (0,0.5,1,2,3h), Insulin release test (0,0.5,1,2,3h); ΔC30/ΔG30, insulin early phase secretion index; AUC _insulin_, area under the insulin curve; HOMA-IR, insulin resistance index; HOMA-β, insulin secretion index; HOMA-IS, Insulin sensitivity index.

### Correlation analysis of HbA1c level with β-HB and islet function

Spearman correlation analysis of all patients included in the study showed that HbA1c was positively correlated with β-HB (r=0.539, P < 0.05), and was negatively correlated with HOMA-β (r=-0.564, P < 0.05), HOMA-IR (r=-0.177, P < 0.05), ΔC30/ΔG30 (r=-0.427, P < 0.05) and AUC insulin (r=-0.581, P < 0.05). In T2DM patients, HbA1c was positively correlated with HOMA-IR (r=0.151, P < 0.05),while in KPD patients, HbA1c was negatively correlated with HOMA-IR (r=-0.254, P < 0.05) (**Table 3**).

**Table 3:** Correlation analysis of HbA1c level with β-HB and islet function (r).

	β-HB	HOMA - IR	HOMA - β	ΔC30/ΔG30	AUC_insulin_
HbA1c					
r	0.539	-0.177	-0.564	-0.427	-0.581
P	<0.001	0.001	<0.001	<0.001	<0.001
HbA1c (T2DM group)					
r	0.155	0.151	-0.505	-0.397	-0.433
P	0.040	0.036	<0.001	<0.001	<0.001
HbA1c (KPD group)					
r	0.261	-0.254	-0.298	-0.184	-0.380
P	0.002	0.003	<0.001	0.031	<0.001

HbA1c, glycosylated hemoglobin A1c; β-HB, β-hydroxybutyric acid; HOMA-IR, insulin resistance index; HOMA-β, insulin secretion index; HOMA-IS, Insulin sensitivity index; ΔC30/ΔG30, insulin early phase secretion index; AUC _insulin_, area under the insulin curve.

### Diagnostic value of HbA1c in KPD

**Figure 2:**
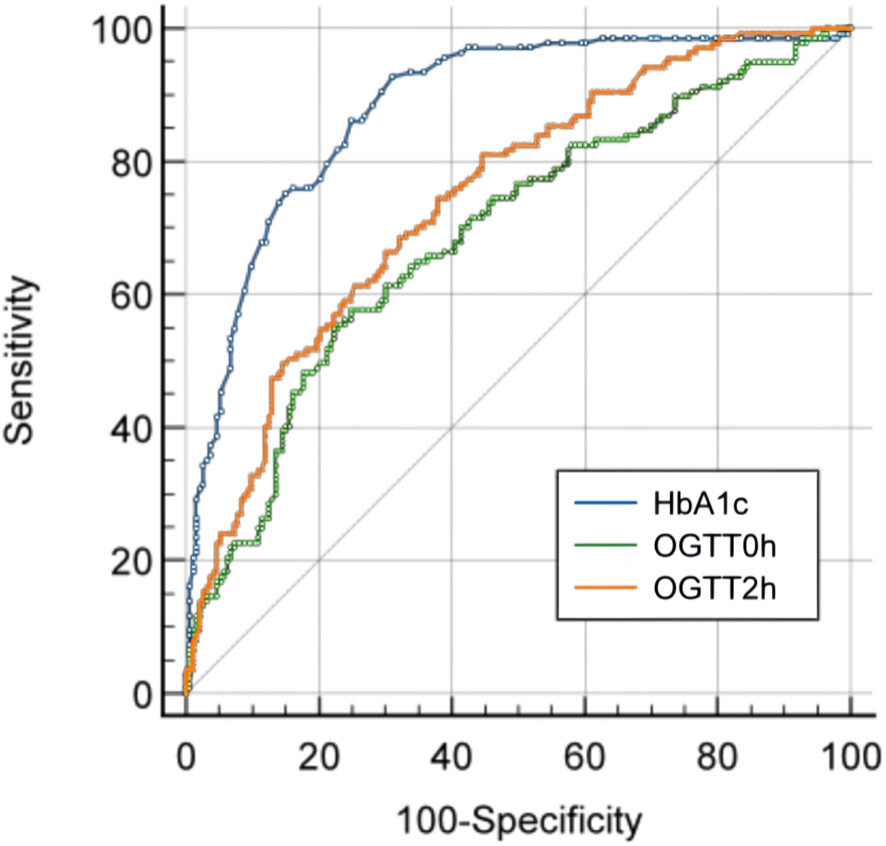
ROC curve of HbA1c, OGTT0h and OGTT2h for the diagnosis of KPD in newly diagnosed T2DM.

ROC curve analysis showed that in newly diagnosed T2DM, the efficiency of diagnosing KPD is the highest, Youden index was 0.616, area under the curve (AUC) was 0.882 (95% confidence interval (CI) was 0.845 to 0.919), sensitivity was 92.70% and specificity was 70.50%. The positive predictive value was 73.26% and the negative predictive value was 81.95%. The positive likelihood ratio was 3.142, and the negative likelihood ratio was 0.104 (**Table 4**; **Figure 2**).

**Table 4:** Sensitivity, specificity and Youden index of HbA1c thresholds in the diagnosis of KPD.

HbA1c(%)	Sensitivity(%)	Specificity(%)	Youden index
10.05	93.40	68.90	0.597
10.15	92.70	70.50	0.616
10.25	90.50	72.00	0.610
10.35	88.30	73.10	0.603
10.45	86.90	73.60	0.600
10.55	86.10	75.10	0.597
10.65	86.10	76.20	0.612
10.85	81.80	78.80	0.590
11.25	75.90	85.00	0.598
11.45	73.70	87.60	0.597

### The diagnostic value of OGTT test for KPD

In all newly diagnosed T2DM patients, the AUC for KPD diagnosed with OGTT0h (fasting blood glucose) was 0.690 (95% confidence interval (CI) 0.632 to 0.748) and the AUC for OGTT2h(2-hour postprandial blood glucose) was 0.745 (95% confidence interval (CI) 0.692 to 0.798) (**Figure 2**). The AUC for HbA1c was significantly higher than the AUC for OGTT0h and OGTT2h, while the AUC for OGTT2h were significantly higher than the AUC for OGTT2h (P<0.05) (**Table 5**).

**Table 5:** Pairwise comparison of ROC curves.

	Difference between areas	Standard Error ^a^	95% Confidence Interval	Z statistic	Significance level
HbA1c~OGTT0h	0.192	0.031	0.132 to 0.252	6.269	P < 0.0001
HbA1c~OGTT2h	0.137	0.027	0.083 to 0.191	5.004	P < 0.0001
OGTT0h~OGTT2h	0.055	0.021	0.015 to 0.095	2.689	P = 0.0072

^a^ The standard error of the area under the ROC curve is calculated using the method of DeLong et al. (24).

## Discussion

This study found a significant association between higher HbA1c levels and the occurrence of ketosis in KPD patients who were newly diagnosed with T2DM. If HbA1c levels are above 10.15%, there is a greater likelihood of developing KPD. The findings suggest that monitoring and predicting the occurrence of ketosis in newly diagnosed T2DM patients can be done promptly based on their HbA1c levels so as to prevent the emergence of diabetes-related acute complications and provide a timely and effective clinical prediction tool for the occurrence of KPD.

This study found that 41.52% of newly diagnosed T2DM patients had KPD, which was consistent with previous research (25). In this study, KPD patients had a younger age of onset, a higher proportion of male patients, and were more likely to be overweight or obese. Additionally, they had significantly lower islet secretion function and required a larger daily insulin dosage to stabilize blood sugar. HbA1c, FFA, and blood sugar levels were also significantly increased in KPD patients compared to T2DM patients without ketosis.

In a study of clinical characteristics of KPD patients, the average HbA1c was 11.3 ± 1.8% (11). Another cross-sectional study of 734 newly diagnosed diabetic patients found that the average HbA1c of KPD patients was 11.56 ± 2.51% (26). The average HbA1c of KPD patients in this study was 12.67%, which was consistent with previous studies. It has been shown that in both T1DM and T2DM, whether in known or newly diagnosed diabetes, HbA1c levels are significantly elevated in patients with ketoacidosis (18). HbA1c reflects the average blood glucose level in the past 8-12 weeks, and it is therefore inferred that ketosis may occur in the context of chronic hyperglycemia, leading to metabolic decompensation through glucotoxic mechanisms that impair β-cell reserves and insulin receptor function (27).

This study divided patients into four groups, A, B, C, and D, based on the quartile levels of HbA1c. The results showed that as the HbA1c concentration increased, the incidence of blood β-HB and ketosis also increased. The incidence of ketosis in the HbA1c quartiles ranged from 8.33% to 82.67%, with a nearly ten-fold increase from the lowest to the highest quartile. These findings suggest that patients with T2DM may be at risk of ketosis in a long-term hyperglycemic environment, and the risk increases with higher HbA1c levels.

The study utilized Spearman correlation analysis to investigate the relationship between HbA1c and various factors. The results indicated a positive correlation between HbA1c and β-HB, but a negative correlation between HbA1c and HOMA-β, ΔC30/ΔG30, and insulin AUC. These findings suggest that as HbA1c levels increase, the secretory functions of islet cells in both early and late stages decrease. Therefore, HbA1c may serve as an indicator of islet function, which is consistent with previous research (28). It is worth noting that with the increase of HbA1c, HOMA-IR also increased. However, when HbA1c levels reached 10.70% or higher, HOMA-IR levels decreased. In the T2DM group, there was a positive correlation between HbA1c and HOMA-IR, while in the KPD group, HbA1c was negatively correlated with HOMA-IR. These results may be related to the decrease of fasting insulin (FIns) at this time, rather than the decrease of insulin resistance.

Current research suggests that ketosis in KPD patients may be linked to a decrease in glucose homeostasis due to prolonged exposure of islet β cells to excess glucose and FFA, which is attributed to the vicious cycle of glycolipid toxicity. chronic hyperglycemia, and lipid metabolism disorders resulting from increased levels of FFA (29). These factors ultimately reduce the function and activity of β cells, leading to a significant decrease in insulin secretion and worsening insulin resistance, ultimately leading to the onset of ketosis (30).

The long-term hyperglycemia environment and the lipid metabolism disorder caused by chronically increased FFA in the body reduce the function and activity of β cell, leading to a sharp decrease in insulin secretion and the persistent aggravation of insulin resistance, ultimately causing the onset of ketosis (26). Each step in the process of insulin synthesis, from gene expression to biosynthesis of proinsulin and secretion of β-cell insulin, plays a crucial role in glucose metabolism in the human body (31). Glucose toxicity can negatively impact any of these processes, ultimately resulting in β-cell dysfunction (13). These pathophysiological mechanisms can cause glucose to continuously accumulate, indicating that glucose toxicity may be a significant contributing factor (32).

This study discovered that patients with KPD had higher levels of FFA, which increased further with higher HbA1c levels. This suggests that there may be an interaction between hyperglycemia and blood lipid metabolism disorders. Glucose utilization disorder can result in a significant increase in the production of FFA which are then used to produce ketone bodies for human utilization (33). In addition, excessive production of FFA in the liver can lead to a reduction in β-cell function and activity, and stimulate gluconeogenesis (30).

Long-term chronic elevation of FFA disrupts the regulation of lipid metabolism, leading to β-cell injury and death through various intracellular mechanisms such as ceramide formation, lipid droplet formation, endoplasmic reticulum stress, oxidative stress, mitochondrial dysfunction, inflammation, and autophagy, etc (34). These mechanisms ultimately result in reduced β-cell function and activity, impaired insulin secretion, and elevated plasma glucose concentrations, creating a vicious cycle (35). FFA exposure for a prolonged period can disrupt glucose metabolism at various stages, resulting in elevated blood sugar levels (36). Therefore, glucose metabolism markers can serve as an indicator for lipid metabolism disorders to some extent (37).

In conclusion, hyperglycemia and severe lipid metabolism disorders can initiate a series of chain amplification effects that damage islet cell function and increase the risk of ketosis. The study found a close correlation between HbA1c and islet function and ketosis, as well as an association with FFA. These findings support previous research on the topic.

According to our study, KPD patients had high HbA1c levels upon admission to the hospital, with an average of 12.67%. Our findings support the correlation between HbA1c levels and KPD, indicating that these patients suffer from chronic hyperglycemia for a long time without noticeable symptoms or timely treatment. Failure to identify and treat such patients can lead to serious complications such as ketoacidosis, coma, shock, and even death (4). Therefore, it is crucial to identify high-risk KPD patients in the early stages of the disease.

Previous studies have not examined the potential of HbA1c levels as a predictor and screening indicator of ketosis in T2DM. Our study aimed to fill this gap, and we found that in newly diagnosed T2DM patients, the highest diagnosis efficiency of KPD was achieved when HbA1c levels were at 10.15%. The Youden index was 0.616.

In this study, we placed special emphasis on high sensitivity in order to better identify patients in the early stages of diabetic ketosis and ketoacidosis. This is due to the severe adverse consequences of these conditions, including death and heavy economic burden during hospitalization. By early identification of individuals in this state, the onset and progression of acute hyperglycemic complications can be properly recognized and prevented.

Our results show that this method has high sensitivity (92.70%) and medium specificity (70.50%), proving its high clinical application value in early identification of KPD high-risk patients. If a patient’s HbA1c level is found to be greater than 10.15% during examination, it is important to monitor their blood sugar, blood and urine ketones more frequently throughout the course of their disease. Appropriate insulin therapy should be actively taken and the dose adjusted accordingly. This will help prevent delays in the patient’s diagnosis and monitoring, and ensure they receive proper treatment (38).

While blood β-HB or urine ketone body detection can determine the presence of ketosis, it is insufficient in identifying high-risk groups for ketosis early on. Additionally, it cannot predict the occurrence of acute complications of diabetes and take preemptive measures in a timely manner. Therefore, there is a need for more comprehensive diagnostic tools to address these issues.

HbA1c is a crucial factor to consider when assessing the level of long-term blood sugar management. It reflects the blood sugar control over the past 2-3 months and serves as a common screening and diagnostic tool for T2DM (39). HbA1c is not affected by transient hyperglycemia caused by stress, nor is it interfered by factors such as stress, diet, exercise, or drugs. It can be checked at any time without fasting or consuming sugar water, making it a better test compliance. Additionally, there is no need for rapid and regular sampling, and blood samples for HbA1c measurement can be kept at 4°C for up to a week, making it a cost-effective and convenient option with high practicality (40). Moreover, with the improvement of the clinical application value of HbA1c, the measurement of it requires strict quality assurance tests, and the determination is highly standardized to meet the standards of international reference values (41).

The OGTT test is a valuable tool for diagnosing diabetes and judging islet function, but it has high requirements and is a lengthy and expensive process. It necessitates regular and rapid sample collection, as well as additional human resources and professional knowledge (42). Patients must fast for at least 12 hours overnight and the test must be conducted in the early morning. During the test period, eating, drinking tea or coffee, smoking, and engaging in strenuous exercise are prohibited. Short-term diet control or physical exercise may also affect the test results (43). If the patient fails to follow the proper regulations for the OGTT test, the procedure will need to be repeated on the following day. Patients with poor islet function are at risk when undergoing this test as it may lead to acute fluctuations in blood sugar levels and potentially cause hyperglycemic emergencies, resulting in artificial secondary damage to the islet function (44). In addition, there are strict requirements on the processing of blood and the preservation of specimens, including rapid separation and storage of plasma and serum at 4°C (45).

However, the high costs associated with the test and poor reproducibility are important factors to consider (46). This study found that the AUC values of OGTT0h and OGTT2h were lower than that of HbA1c, indicating that HbA1c has better diagnostic performance for KPD than OGTT test.

This study highlights the clinical significance of HbA1c, which has a strong correlation with islet function and blood β-HB. The high sensitivity of HbA1c in diagnosing KPD can aid in the early identification of high-risk groups and prevent disease progression. It should be noted that this study has limitations in terms of its scope and methodology. Specifically, it is a single-center retrospective study of hospitalized patients, and the sample size is limited. Therefore, further research with a larger sample size is necessary to draw more definitive conclusions.

## Conclusion

In summary, for newly diagnosed T2DM patients, HbA1c > 10.15% was associated with a greater likelihood of developing KPD. In clinical practice, timely and active examination for the presence of ketosis should be conducted and treatment measures such as islet hypoglycemia should be promptly taken to prevent the occurrence of KPD and further deterioration of the disease.

## Data availability statement

The original contributions presented in the study are included in the article/**Supplementary Material**. Further inquiries can be directed to the corresponding authors.

## Ethics statement

The studies involving humans were approved by the Ethics Committee of Wuhan Fourth Hospital. The studies were conducted in accordance with the local legislation and institutional requirements. Written informed consent for participation was not required from the participants or the participants’ legal guardians/next of kin in accordance with the national legislation and institutional requirements.

## Author contributions

RM and BP contributed equally to this manuscript, and BP is the co-first author of this article. RM conceived and designed the study, analyzed the data, and drafted the first manuscript. BP, YX were involved in the collection of the data and preparation of figures and tables. BP critically revised the manuscript. All authors contributed to the article and approved the submitted version.
